# Canadians’ trust in government in a time of crisis: Does it matter?

**DOI:** 10.1371/journal.pone.0290664

**Published:** 2023-09-08

**Authors:** Hoda Herati, Kathleen E. Burns, Maria Nascimento, Patrick Brown, Michael Calnan, Ève Dubé, Paul R. Ward, Eric Filice, Bobbi Rotolo, Nnenna Ike, Samantha B. Meyer

**Affiliations:** 1 School of Public Health Sciences, University of Waterloo, Waterloo, Canada; 2 Department of Sociology, University of Amsterdam, Amsterdam, The Netherlands; 3 School of Social Policy, Sociology and Social Research, University of Kent, Canterbury, United Kingdom; 4 Laval University, Québec, Canada; 5 Research Centre for Public Health, Equity and Human Flourishing, Torrens University, Adelaide, Australia; 6 Department of Parks, Recreation and Tourism Management, North Carolina State University, Raleigh, North Carolina, United States of America; The University of Alabama, UNITED STATES

## Abstract

The ability of governments and nations to handle crises and protect the lives of citizens is heavily dependent on the public’s trust in their governments and related social institutions. The aim of the present research was to understand public trust in government during a time of crisis, drawing on interview data (N = 56) collected during the COVID-19 pandemic (2021). In addition to the general public (n = 11), participants were sampled to obtain diversity as it relates to identifying as First Nations, Métis, and Inuit (n = 7), LGBT2SQ+ (n = 5), low-income (n = 8), Black Canadians (n = 7), young adult (n = 8), and newcomers to Canada (n = 10). Data were coded in consideration of social theories of trust, and specifically the nature of trust between individuals and institutions working with government in pandemic management. Canadians’ trust in government was shaped by perceptions of pandemic communication, as well as decision-making and implementation of countermeasures. Data suggest that although participants did not trust government, they were accepting of measures and messages as presented through government channels, pointing to the importance of (re)building trust in government. Perhaps more importantly however, data indicate that resources should be invested in monitoring and evaluating public perception of individuals and institutions generating the evidence-base used to guide government communication and decision-making to ensure trust is maintained. Theoretically, our work adds to our understanding of the nature of trust as it relates to the association between interpersonal and institutional trust, and also the nature of trust across institutions.

## Introduction

The ability of governments and nations to handle crises and protect the lives of citizens is heavily dependent on the public’s trust in their governments and related social institutions [1]. According to Llewellyn [2], “in times of crisis, trust is the most important thing to consider if you want to communicate health advice.” Indeed, citizens’ willingness to adhere to implemented countermeasures during a pandemic is greatly impacted by their trust in politicians, scientists, and, institutions [1,3]. The aim of the present research was to understand public trust in government during a time of crisis through the analysis of interview data collected during the COVID-19 pandemic (2021). Drawing on social theories of trust, we also speak to the nature of trust in individuals and institutions working with government in pandemic management.

## Background

Trust is said to occur at both institutional and interpersonal levels, with trust in individuals to some extent impacting trust in the organization they represent, though it remains disputed in who or what trust comes first [4]. Herein we focus on institutional trust in government; that is, trust that is based on the perceived beneficence, competence, and integrity of government as an institution [5]. Our focus in the present paper is institutional trust and specifically, that which is placed in government in a time of crisis. Institutional trust has been described as “the extent to which individuals accept and perceive institutions benevolent, competent, reliable, and responsible toward citizens” [6,7]; a definition that points to the dimensions of care and competence as they factor into one’s assessment of whether to trust an institution. Indeed, research has demonstrated that public trust in political institutions, and their representatives, is predicated on public perception that the actions and intentions of these institutions are in the public’s best interest and deliver results that meet their expectations [8–10].

In times of crisis, such as the COVID-19 pandemic, public trust in government agencies, public health officials, the healthcare system, and their representatives is essential. Public trust in government and medical authorities, for example, has facilitated greater acceptance of public health measures put in place to stop the spread of COVID-19 –e.g., social distancing [11] and vaccine uptake [12,13]. Public policies that are reliant on public cooperation with policymakers are therefore more likely to be successful if the public trust those generating and implementing such policies [14,15]. For example, research suggests that higher trust in government is associated with reduced COVID-19 mortality rates [16]. The association between trust in government and COVID-19 mortality rates is likely mediated by countermeasure acceptance and adherence, whereby trust positively influences countermeasure acceptance and therefore COVID-19 mortality rates in the population.

As a result of COVID-19, the Canadian government, like most nations, was placed in an unprecedented position, generating measures to contain the spread of disease, flatten the curve, and maintain the integrity of their healthcare systems [17]. Social gatherings, in-person attendance at work/school/universities, engagement in sports and leisure activities, and traveling were subject to rigid restrictions under these countermeasures [18]. However, support for such measures could not be assumed and particularly as the pandemic progressed, nations became polarized in their level of support for, or opposition towards, government measures. Indeed, Canadians’ trust in government has been challenged by the COVID-19 pandemic, economic turmoil, and a demand for equality and stability. According to the 2021 Edelman Trust Barometer [19], trust in the Canadian government declined by 11% between May 2020 and January 2021. The ever-changing nature of COVID-19, government’s contradictory and confusing information (e.g., details regarding the accepted size of social gatherings [20]), and pandemic-related stress and anxiety can negatively impact the public’s trust in government and may help to explain this decline over time [21]. Indeed, psychological factors as potential sources of distrust in social institutions are important considerations when developing strategies to (re)build trust as it relates to the acceptance of government health recommendations. For example, Lieberoth et al. (2021) found that individuals trusting of government efforts to fight the COVID-19 virus experienced less stress and greater compliance of behavioural guidelines [22]. Other psychological factors have been found to be associated with trust in the government. Particularly, conspiratorial thinking has been described as a predictor for reduced trust in government, while anti-expert sentiments have been found to be a predictor of reduced trust in science [23]. Although variability in these associations exist across countries, a broad understanding of these psychological factors can help with understanding the decision-making process of individuals during the COVID-19 pandemic when opting (or not) to comply with public health measures.

Literature [24] suggests that a proactive and timely response to COVID-19 may be also associated with the maintenance of public trust during the pandemic. Criticism of the Canadian government may therefore be related to perceptions of poor management of the pandemic. According to Tang et al. [25], despite Canada’s prior experience with SARS outbreaks in 2003, the country’s response to the COVID-19 pandemic was relatively slow and insufficient. The government’s hesitancy to close the international borders and insufficient personal protective equipment supplies as a result of sending 16 tonnes to China early in the pandemic, were indeed among the reasons for Canada’s inability to sufficiently respond to the initial threat of COVID-19 [25].

Criticisms of the Canadian government have manifested in the rejection of measures by numerous Canadians, showing vehement lack of support for government among a small but highly visible minority. In late January 2022, the so-called ‘Freedom Convoy’, a group of Canadian truckers held rallies that posed a sufficient threat to public peace and safety, widely considered unlawful. Their goal was to demonstrate their rejection of COVID-19 vaccination mandates and restrictions in the nation’s capital, Ottawa, and other cities across the country [26]. This demonstration, among others, arguably demonstrates a distrust of government, fuelled by their response to the pandemic and consequential impacts on the health and financial wellbeing of Canadians. Notably, there is a need to acknowledge that trust in social institutions varies across social groups. For example, populations with experiences of marginalisation, systemic oppression, and discrimination are less likely to trust the government [27–29]. As such, some individuals do not trust government due to perceived inaction or poor decision-making relating to specific events (i.e., COVID-19 pandemic and vaccine mandates), while a lack of trust for marginalised populations may relate to experiences of harm related to structural inequities that have yet to be addressed (e.g., [46]).

Within the present study, we conducted interviews to identify public perceptions of the government’s response to the pandemic, specifically exploring how their perceptions related to trust in government as an institution. In our investigation of institutional trust, however, we are cognisant of studies demonstrating a positive correlation between interpersonal and institutional trust [30–33]. Critical to this study was therefore also an exploration of the foundations of trust in government, as scholars remain divided with regard to whether one must first trust in the representative before the institutions, or whether the opposite must occur [34]. We also approach the data with consideration of how trust in one social institution (e.g., the federal government) influences trust in other social institutions (e.g., public health), as evidence would suggest distrust in government would negatively impact trust in other social institutions affiliated with government [35]. This information provides insight regarding in whom or what trust needs to be (re)developed or built to generate support for government initiatives aimed at population health. We seek to document the nature of public trust in government during a time of crisis and situate our data within existing literature that might guide strategies for (re)building and/or maintaining public trust in government in a time of crisis.

## Materials and methods

The data presented herein are drawn from a larger research project exploring the role of trust in Canadians’ acceptance of COVID-19 countermeasures. In this article, we focus on interview questions specifically investigating the nature of trust in government during a crisis, considering public perceptions of the government’s response to the COVID-19 pandemic in Canada. We asked participants about their experiences during the pandemic, including their acceptance of public health measures, the perceptions of how the government has handled the pandemic, and specifically, their trust in both the provincial and federal governments.

Semi-structured interviews (N = 56) were conducted by author HH with members of the general population (n = 11) and purposely sampled sub-populations historically disadvantaged by social institutions in Canada to obtain insight into the perspective of those most likely to experience distrust: First Nations, Métis, and Inuit (n = 7), LGBT2SQ+ (n = 5), low-income (n = 8), Black Canadians (n = 7), young adult (n = 8), and newcomers to Canada (n = 10).

Participants were recruited and interviews were conducted between August and December 2021. Participants were recruited through Leger, Canada’s largest and most representative research marketing firm to gain representation from harder-to-reach populations. Leger recruited potential participants and provided contact information to the research team.

Interviews were conducted by phone or via virtual platforms (Cisco Webex, Zoom, or Microsoft Teams), depending on the participant’s preference. Interviews were audio recorded and transcribed verbatim. Memos served as a record of the researcher’s initial thoughts on each interview for the purpose of communicating the analytic progress to the team and for recall later down the process of analysis.

Initial coding required researchers to work systematically through the entire dataset, giving full and equal attention to every data point. For this exploratory phase, we were open to coding all data before determining what was or was not significant to the analysis [36]. *In vivo* codes (the participants’ own words) were used to help preserve participants’ meanings of their views and actions. Focused coding involved taking earlier codes that continually reappeared and using them to organize large amounts of the data into meaningful themes. During focused coding, the analyst built and clarified themes relevant to the research aim of documenting the nature of trust in government. Finally, we approached the data themes in consideration of social theories of trust [34,37], and specifically trust in government as it relates to trust within and between individuals and institutions involved in pandemic management. To achieve coding-reliability, the team met weekly to discuss ongoing analyses and themes (initial and focused coding) for a period of four months. At least two members of the team reviewed transcripts, and during the interview process, memos were shared with the larger team. All coding for the interviews were completed using NVIVO coding software.

Ethics approval was obtained by the University of Waterloo Research Ethics Committee before the study began (project # 42486). Researchers were provided with information that could identify individual participants during or after data collection. However, this information was only available to individuals listed on the ethics application and remains confidential on a password protected computer. All participants provided written or oral consent for the recording and use of quotes in publications. Pseudonyms have been used to maintain anonymity.

## Results

Participants were representative of most Canadian Provinces, with Ontario having the highest number of participants (45%). The gender distribution of the participants was 64% female, 34% male, and 2% non-binary. For recruitment purposes, individuals with annual incomes <$40,000 annually (CAD) were considered low-income (LI), those who had lived in Canada for less than 5 years as newcomers (N), and individuals aged 18–24 as young adults (Y). Furthermore, we included participants who identified as black Canadians (BC), First Nations, Metis, and Inuit (FNMI). Regarding sexual orientation, 84% of the respondents were heterosexual, 14% were LGBTQS2+ (LGBT), and 4% preferred not to respond. Most participants (38%) preferred not to disclose their political affiliation. Participant characteristics regarding the mentioned sub-groups can be found in Table 1.

**Table 1:** Participants’ demographics summary.

Variable		Freq (%)^a^
**Age**	18–34	25 (44.6)
	35–54	16 (28.6)
	55–74	12 (21.4)
	>75	3 (5.4)
**Province**	Alberta	11 (19.6)
	British Columbia	5 (8.9)
	Manitoba	6 (10.7)
	New Brunswick	3 (5.4)
	Ontario	26 (46.6)
	Prince Edward Island	1 (1.8)
	Quebec	3 (5.4)
	Saskatchewan	1 (1.8)
**Gender Identity**	Man	19 (33.9)
	Woman	36 (64.3)
	Non-Binary	1 (1.8)
**Sexual Orientation**	Heterosexual	46 (82.1)
	LGBTQS2+	8 (14.3)
	Not Disclosed	2 (3.6)
**Years in Canada**	Not Applicable	37 (66.1)
	≤ 5 years	4 (7.1)
	>5 years	15 (26.8)
**Household Income**	≤ $19,999	2 (3.6)
	$20,000 - $59,999	20 (35.7)
	$60,000 - $99,999	17 (30.4)
	$100,000 - $139,999	11 (19.6)
	≥$140,000	4 (7.1)
	Not Disclosed	2 (3.6)
**Federal Political Party Supported**	Liberal	14 (25.0)
	Conservative	9 (16.1)
	New Democratic Party	6 (10.7)
	People’s Party of Canada	5 (8.9)
	Green Party	1 (1.8)
	Not Disclosed	21 (37.5)

^a^Percentages may not sum to 100 due to rounding.

Two broad themes were identified as relevant to the nature of citizen trust in government: pandemic communication, and decision-making and implementation of measures. We attribute quotes presented below to individuals based on their identified sub-groups (e.g., LI, LGBTQ, N, Y, BC, FMNI) though our goal is not to look for differences across subgroups. Rather, data from the entire sample (e.g., general population and sub-groups) were examined together to give a comprehensive understanding of perceptions of the government’s response to the COVID-19 pandemic. However, we also include notation related to political affiliation–that is, if they support political parties representing left of centre (NDP, Green, Liberal) and right of centre (Conservative, PPC), though 37.5% chose not to disclose.

### Pandemic communication

Participants largely accepted and trusted evidence-based messages communicated to the public by experts and scientists. They also stressed the importance of delivering clear, consistent, and transparent messaging during a public health crisis, tailored to diverse populations, to build and maintain trust in government agencies.

#### Trusting the information generated for government

With regards to communication, participants mentioned that they were likely to trust information provided by professionals and scientists they deemed to be credible, and not necessarily the political parties.

“*A guideline is a guideline*. *A guideline is made by the professionals*, *not political*.*”–GP7*

Interviewer: “And why are you more trusting of medical authorities than government?”; “Because they have the science behind them.”–LI4 (Liberal)

Participants noted that the government’s messaging should be informed by evidence and experts’ opinions, the sources of information they trusted more during a crisis. In these accounts, there is a noted relationship between scientists and government, with the former generating the evidence and the latter being the ones to communicate the information.

“*Personally*, *the most credible source of information is going to be peer-reviewed literature*, *so journals*. *Then experts*, *like medical experts*, *just because they have the expertise to be able to interpret that literature and typically*, *they do make decisions based on that literature*, *so that would be the next source*. *And then government*, *just because they typically*, *at least in the pandemic*, *make decisions based on that evidence sooner or later*.*”–Y3 (Liberal)*

“*I know that even though the government are the ones announcing it*, *I know there’s professionals who work on those plans behind the scenes*. *And they’re putting out the message*, *but I think that they’re not the ones creating those measures necessarily*. *There’s a team behind them that I do trust too*.*”–Y5 (Liberal)*

It was suggested that government should work to a greater extent with the health field and professionals in a way to communicate reliable information, enhance the public’s knowledge about the pandemic, and overcome misinformation.

“*I think especially wrong information is from the non-scientific resources*. *But seriously I don’t know how the government can control that conspiracy or misinformation*. *But these things really did need some control at least*, *the scientific or actual [people] like from the medical field*, *they need to come forward*. *Our government needs to take their spot or use them [to increase] awareness*. *And the awareness*, *needs to inform people how they should behave even after like vaccination and precautions or measurements*. *Yeah*, *they need to follow even after later*.*”–GP5 (NDP)*

#### Clarity and consistency in communication

Participants indicated that the lack of clarity and consistency in communication with the public during COVID-19 led to uncertainties about the messages disseminated and countermeasures implemented during the pandemic.

“*There’s been a lot*. *It’s been really annoying how often they change*, *and that can make it difficult to understand what*, *we as citizens are expected to do because they keep changing*, *and it also makes us doubt the research that’s going into this*, *because it seems like the research is constantly changing as well*.*”–GP4 (Liberal)*

“*They were communicated fine*, *but as I said*, *they kept altering them*. *When they were communicated*, *they were done well*, *but a few days or weeks would go by and then there would be a change in it*. *And you got to be careful with that*. *If you’re going to say something’s useful*, *or valuable*, *or relevant to you*, *you can’t keep changing it*. *But in the communication*, *yes*. *It was fine*.*”–GP11 (PPC)*

Despite the noted trust in scientific experts and peer-reviewed research, there was frustration with the changing nature of evidence. In the above quotes, however, the criticism seems to fall on the messengers (the government) rather than those behind the messages. Y3 spoke to the changing of information, but again notes that government action ‘exposed’ the government’s level of involvement in public health measures.

“*[The] government allowed police forces to enforce stay-at-home orders*, *and then the very next day or within 48 hours they recalled that*… *So*, *the back and forth*, *flip flopping between what these public health measures look like and exposing how much politics is involved in the public health measures*.*”–Y3 (Liberal)*

Participants also stated that the government needs to communicate with the public considering the diversity of the audience. For example, the need to consider citizens’ literacy levels when creating and delivering messages about the pandemic was emphasized.

“*The communication could be clearer*. *And not to be rude or whatever but dumb it down a bit so everybody can understand it*. *Yeah*, *because I think sometimes maybe the wording isn’t easy enough for some people to get*.*”–LI2*

Further, participants spoke to the importance of considering societal factors such as education level, age, and socioeconomic status when communicating about the pandemic to all citizens.

“*More information is always good and communicating as much as they get here may have helped them more*. *Education levels back in the rural areas are slightly less than they are in the suburban areas where I am*, *so that might have an impact on it too*. *Socioeconomics are slightly less there than they are here*, *that may have an impact on it*. *There’s probably lots of factors*, *but yeah*, *the level of communication was a big difference for us to like seeing where our parents are and where we are it’s different*.*”–GP1 (Liberal)*

“*You’re dealing with people who are a bit more educated in terms of science*, *they can understand that the information has changed*, *you’re learning more every day*, *but there’s people who don’t have that capability*.*”–GP3 (Liberal)*

Participants also expressed that the federal, provincial, and municipal governments must collaborate to prevent discrepancies in taking decisions that could confuse the public. The participants shared frustrations that the leaders of federal, provincial, and municipal governments had different approaches to dealing with the pandemic instead of following the same directives.

“*At the beginning*, *it was a little touch and go*. *I mean*, *like the prime minister would say one thing the premier would say another thing*, *and the mayor said he would say something else*. *So*, *I think you have to listen to them all and try to wage each decision and try to act accordingly*.*”–LI3 (Liberal)*

#### Transparency, openness, and honesty in communication

Participants noted a lack of transparency in terms of government sharing of information, not limited to pandemic management. Rather, withholding information from the public was noted as a long-term issue that has affected public trust in government.

“*I think having them be pretty clear as to where our resources are going and*, *I guess having that information be available*. *I can do my part more on researching that and looking it up but having those types of things be available to people who want to access them to see*, *well*, *how our*, *you know*, *our tax dollars being used in health care because*, *you know*, *we’re struggling*. *The health care system is struggling to keep up with lots of things even before the pandemic and so this was just kind of*, *I think*, *exposed some areas that need to be addressed*. *A transparency*, *I guess would be a good word for that*.*”–GP10*

“*I think it’s circling back around to transparency as a system*. *I think this is an age of pessimism*. *I think it’s an age of distrust in what is simply being told to you*, *regardless of how credible the source*. *I think people like to see and have different conversations and transparency in the information that they don’t feel that information is being withheld or anything like that*.*”–LGBT5 (NDP)*

Participants also suggested that the government authorities at times deliberately withheld information from the public, leading them to question the interest being served in communication.

“*I feel like the government is not necessarily working against people*, *but I think they have an obligation to tell the community*, *society*, *what they need to know and what they want to hear*. *Not necessarily actual facts…it’s like they [the government] mask information certain ways*, *certain things they say*, *messages…kind of like smoke and mirrors*. *Just like you’re talking about one thing to avoid another thing*.*”–BC3 (NDP)*

### Decision-making and implementation of measures

A second major theme in the data was considerations of trust in government as it related to the implementation of measures. Participants spoke to several (in)actions of government that influenced their trust, namely: delayed response to pandemic; prioritization of political agenda over public health; inefficient allocation of medical resources and supplies; inequities in health services provision and failure to consider equity with regards to the economic welfare of citizens and COVID-19 related care. Trust was also negatively impacted by government action considered to restrict personal freedoms.

#### Delayed response to the pandemic

Participants were critical of the government’s lack of action early in the pandemic to mitigate spread. Some participants shared concerns regarding what they saw as an early easing of mandates and measures.

“*I think government doesn’t need to stop yet and say*, *"Relax guys*. *It’s over*.*" It’s not over*. *We need to be sure government is taking care of it very well and continue to do good job and be very strong*. *So*, *I think it’s important*.*”–GP6*

“*I think that they’re not strong enough*. *I think the country shouldn’t be afraid of closing down for five or six days*, *and that’s always worked*. *But they’re not doing that anymore*. *A full closure*. *So be it*.*”–LI6 (Liberal)*

“*I think they could have done a better job of keeping the border closed and keeping Canadians in Canada*. *And I would like to think that would have maybe stopped these variants from coming in*. *So*, *I’m a little disappointed at that part*.*”–LI2*

#### Prioritization of political agendas above public health

Trust was said to be challenged when government was seen as putting their interests ahead of the public. For example, following the second wave of the pandemic, the then-leader of the federal government called for an early election which was cited by some as unusual timing.

“*So something that everyone is upset about is that when the Liberal Party first won the federal election a couple of years ago*, *they still haven’t followed up on what they were saying they would do in the campaign*, *but is quite odd*, *and then right now*, *calling a federal election early seems like a strange move as well*, *and I can’t see how that benefits them*.*”–GP4 (Liberal)*

The following also speaks to questions about whose interests are served. Again, the perception is that the public health messages were communicated well but the nature of government involvement negatively impacted trust. Importantly, as above, the criticism here is not of the scientists or individuals behind government messaging but rather, government as an institution.

“*Overall*, *things have been well communicated*. *Just coming back to that idea of the politics of it*, *I think the politics have really muddled and interfered with that clear communication*. *So*, *I think in that respect*, *I think the public health measures themselves*, *when they were announced was done well*. *But*, *the build up to that fostered this sense of mistrust*.*”–Y3 (Liberal)*

#### Inefficient allocation of medical resources and supplies in a pandemic

Participants suggested that insufficient healthcare funding, resources, and staff before and during the pandemic contributed to the lack of trust in both the federal and provincial governments.

“*In the last however many decades I haven’t had a lot of faith in political leadership at the provincial level to give the medical system the money that they need to do their job*. *I don’t have a lot of faith in government at the federal level to provide money to change the health transfer systems to the provinces*, *to give the provinces the money they need to give the hospitals the money they need to provide the services”–GP1 (Liberal)*

GP1 notes a lack of faith in both federal and provincial government in providing what is needed at the health system level. Respondents mentioned that health and social funding have always been an issue in terms of provision of sufficient resources in Canada, but this became more apparent during the pandemic.

“*I think that a lot of this is on the shoulders of the government who hasn’t done their job in the last 20 years*. *And not putting the correct resources*, *not bringing enough people to support the system*. *When people are waiting 500 days… In my area of Montreal*, *people are waiting 500 days to try to find a general practitioner*. *There’s wait lists that are 500 days*. *I don’t think that’s acceptable*.*”–GP3 (Liberal)*

It was also stated that the government needs to improve the efficiencies and effectiveness of resource use in the healthcare system and other public sectors.

“*Because it seems to me that Covid highlighted inefficiencies and deficiencies in the senior care system*. *So*, *there I suppose maybe my belief in Canada’s health care did take a little bit of a nosedive*.*”–LI4 (Liberal)*

In the decision-making process regarding pandemic measures, participants stressed the importance of setting priorities during a time of crisis.

“*I think it’s a combination of funding because*, *I mean*, *at the end of the day*, *everything costs money*. *And I think that’s where a lot of those decisions get made of what is important and what isn’t*.*”–LGBT4 (Conservative)*

Additionally, participants expressed dissatisfaction with how the government provided supplies to other countries during the early phase of the pandemic while the threat of COVID-19 was growing in Canada. This act was considered by some to be inappropriate as the federal government has an obligation to provide enough supplies for Canadian citizens during a pandemic to avoid shortages.

“*They should have been taking care of Canada first*. *When it first started*, *the federal liberal government gave to countries in need a lot of the supplies that Canada had*, *and then we had shortages in supplies*. *So*, *the federal government didn’t look at Canada first*, *they were looking at (…) global politics*.*”–LI1*

#### Inequity in health services provision

Regarding the government’s pandemic response, participants expressed dissatisfaction with perceived disparities in funding, services, and support between different (i.e., urban vs. rural) communities. They argued that citizens should have access to services regardless of their geographic location.

“*I would trust the system to provide the same level of care no matter where I was*, *no matter what happened*, *who the doctor was*. *I know because of individual differences people wouldn’t be all the same*, *so they’ll be differences in how the service is provided and there may be funding differences because of where I’m getting services*, *but I hope for a decent level of care no matter where I am in the system*. *But other than the things where I’ve outlined different trust levels between rural and urban doctors*, *I can’t think of too much more that I haven’t said in that regard*.*”–GP1 (Liberal)*

“*To have available the care that you need no matter where you are*. *I think to have the same level of care for*, *say*, *one more sparsely populated areas that they have with urban settings*, *that the care encompasses everyone*.*”–LI4 (Liberal)*

“*I think just providing equitable but also culturally competent care so when interacting with the*

healthcare system, just an understanding that, or just an openness to understanding where I’m

coming from, and how things might be different between patients and treating all patients

equitably, regardless of identity features.”–Y3 (Liberal)

Concerning the relationship between political interests and public health concerns, participants expressed frustration with government based on the perception that they did not provide equal care and attention to all provinces across Canada. In their view, those provinces with more voting power have received better care than the others.

“*(*…*) the federal government*… *isn’t really worried about Manitoba because it doesn’t have an influence over voter*… *or who’s in power*. *There’s less attention given to the province of Manitoba (*…*)*. *More the liberal because they had less members of parliament from the west*.*”- LI1*

Additionally, they stressed the importance of providing better communication and more accessible resources regarding the pandemic in rural areas, like those provided in urban areas. The participants believed that this would make rural residents more aware of the risks of the pandemic and more likely to follow the implemented countermeasures.

“*My wife and I go back to where our parents are in a rural setting quite regularly and there’s a large group there*, *like their vaccine levels are probably 10–15% less than they are here in our city*, *and there’s a very quiet group there*, *they’re not the protesting kind*, *but there’s a kind that’s just saying*, *"You know what*? *I’m not getting the vaccine*. *I don’t need it and I don’t want to risk it*. *I’m 70 years old or I’m 60 years old and I’m fine*.*" They’ve only had one case in the little town where they are in the whole time and they haven’t been personally impacted by it*, *so they don’t see the threat that it is*. *So there is that quiet group back there*, *just that’s my personal experience*, *is that that group is there and it’s large and when you see the vaccine numbers*, *you go*, *"Oh*, *God*, *yeah*.*" It is a big number of people*.*”–GP1 (Liberal)*

#### Failure to consider equity with regards to the economic welfare of citizens

Participants generally believed that the Canadian government did not strike a proper balance between containing the spread of the virus and addressing the economic impacts of this global pandemic. Using the example of travel restrictions, participants spoke to the importance of easing travel or opening businesses to address economic concerns. However, they saw a need in other cases for more restrictions.

“*Some places you could not go into*, *they were closed*. *And I thought that was a bit unfair to the owners of those*, *but I agreed with it*. *But some of them have been reopened again*, *and yet we still got high numbers so it seems to me we needed to have closed them longer*. *I don’t think they should have been opened as fast as they were*.*”–GP11 (PPC)*

“*Well*, *Americans as a whole have a lower vaccination rates and very significant pockets of the country that are refusing COVID measures*. *We are allowing Americans to cross the border at a time when Americans have deemed Canadians crossing the border to be a danger*. *So if Canadians going south are a danger to Americans*, *I don’t see how Americans coming here isn’t a danger to Canadians*?*”–GP2*

“*I think they have to close the border and check every airplane in the airport*.*”–GP7*

Furthermore, participants believed that the pandemic restrictions were unfairly applied. For example, they noted that small businesses had tighter restrictions compared to big box stores, affecting local and small businesses. They suggested engaging these small businesses in the decision-making process.

“*Big concern is that they were applied unevenly*. *Small*, *locally owned businesses were forced to close completely or only do curbside pickup*, *while big box stores and large corporate entities were allowed to continue to do business*, *and that created unnecessary very unfair stress and trauma locally their business*.*” GP2*

Another example of inequities as they relate to the economic impact on citizens is the government’s provision of financial assistance. Participants suggested that this was not evenly distributed among different groups of people from different communities. The unfair allocation of financial support was mainly mentioned in reference to seniors. These individuals suggested that certain communities such as low-income may have little trust in government institutions due to a lack of communication between government officials and these communities.

“*That seniors in Canada*, *they were*… *They looked at a certain level of seniors and even giving them money when they were giving them COVID funds that our people survive*. *They only looked at the oldest years*, *which didn’t affect me*. *75 and older got a couple lump sums of money*, *and because I’m 73*, *I didn’t get it*. *My boyfriend did*, *but I didn’t*. *And a lot of the seniors in Canada suffered due to that*.*”–LI1*

## Discussion

During the pandemic, a period of immense uncertainty, the public were asked to trust the official guidance of Canadian social institutions–to trust that the right thing was to stay home, vaccinate, physically distance, and forgo leisure and in many cases, their livelihoods. To foster acceptance of official guidance, institutions and officials representing these institutions continue to consider how to build trust, assessing the consequences of broken trust, and identifying what can be done to restore trust. Indeed, the decline in the overall trust in political leadership in Canada [38] threatens the legitimacy of government as an institution in Canada and relevant to the health of the population, acceptance of recommendations moving forward to protect the health of the public. As such, the present study sought to investigate Canadians’ trust in government during a period when trust was arguably challenged more than ever before. Our results suggest that public trust is highly dependent on government communications and implementation of measures during a pandemic. In the following section we speak to these two primary themes before speaking to the contributions of this work in terms of understanding the nature of trust between individuals and institutions working with government in pandemic management.

### Trust as it relates to government communication and implementation of measures

Criticism of government was discussed in relation to inequities in pandemic communication (e.g., communication needed to be tailored to specific population groups) and inconsistencies in messaging. This finding is consistent with prior research whereby unclear and inconsistent messaging from government institutions has been associated with criticisms of government competence [5], a core dimension of trust. Akin to other research [5,14,39–41] we found that concerns over transparency (“politics have really muddled and interfered with that clear communication”) and honesty (“telling people what they want to hear”) were found to impact trust in government. Criticisms regarding inaction to address inequities (or indeed, exacerbating inequities) relates to a core dimension of trust, beneficence, and also literature regarding the importance of taking into account the needs of various subpopulations in pandemic management [9,14]. These data may speak to the values of participants–that of social welfare–which was, at the time of data collection, stated to be at the core of federal leadership. To foster trust during a crisis, government messages must be based on citizens’ values and our data suggest that the values of our participants ran counter to values underpinning government (in)action.

In addition to communication, trust in government is heavily influenced by the government’s preparedness, decisions, and performance during a pandemic. Indeed, trust in social/political institutions is the result of the actual performance of the institution–their competence [30,33,42–44]. Certainly, our data suggest that there were questions about the competence of the government in pandemic management, a key dimension of trust. Research has found that in times of crisis, citizens’ trust in their governments can be maintained by a proactive and early response and can be undermined if the government fails to act timely and properly during the pandemic [24]. Our participants perceived the government’s response to the pandemic to be relatively slow (e.g., slow to close international borders) and inadequate which negatively impacted their trust in government. The government’s (in)action was also said to be related to the government prioritizing political agendas over public health—the belief that actions and intentions were not in the interest of citizens likely fuelled the erosion of trust, threatening the government’s perceived integrity [8,9]. The perceived in- or late-action of government led to additional questions regarding the competence of government (e.g., regarding the provision of health services) which is critical to trust in government [5,45].

As noted, trust differs across populations. Within disadvantaged or marginalised populations there are unique factors that challenge trust and related acceptance of countermeasures. Our sample included participants who self-identified as belonging to Indigenous (First Nations, Métis, and Inuit), LGBT2SQ+, and racialized communities. These population groups have been marginalised, oppressed, and discriminated throughout history by both the governments and the healthcare systems, and these issues prevail to this day [27–29,46–50]. These forms of harm likely led members of these communities to question the integrity and intentions of government and public health, and the interests served by government mandates. Indeed, our data suggests that some of the factors driving a lack of trust relate to the failure of the government to support Canadians at large. Particularly, the government were criticized as failing to address and acknowledge systemic issues that further disadvantaged marginalised populations within Canada. Indeed, inequities with regards to resource allocation were also said to impact trust in government, as it related to integrity. Concerns regarding equity in distribution became reality in Canada and globally, as evidenced by a vast body of research noting significant differences in COVID-19 cases and death rates among disadvantaged populations [51,52]. For example, fewer medical staff and medical supplies were available during COVID-19 in disadvantaged communities in rural areas with limited space, staffing and supplies [53]. These inequities also played out in communities unable to follow measures due to living and working conditions [54,55]. Demand for medical supplies, capacity and staff has been a global concern, as most countries face resource shortages. These shortages can negatively impact citizens’ trust in their governments, given the role of government agencies in healthcare regulations and policies [56]. To maintain trust, governments might establish protocols including the development of a contingency plan to efficiently allocate medical resources, particularly in communities at greatest threat, in the case of a health crisis and be prepared to combat scarce hospital supplies and medical staff [57].

### The nature of trust during a time of crisis

COVID-19 created a space in which to investigate public trust where arguably the awareness of trust in institutions has never been more salient. Theoretically, our work adds to our understanding of the nature of trust as it relates to the association between interpersonal and institutional trust, and the nature of trust across institutions.

Theory would suggest that the assessment of whether to trust messages from scientific or health institutions (e.g., Public Health Agency of Canada and Canadian government-funded research institutions) would relate to whether one trusts the institutions with which they are affiliated (federal government) [34]. Further, it would be expected that given individuals behind government messaging (e.g., scientists and public health officials) are part of larger institutions, trust in these individuals might extend to the institutions within which they operate or be an extension of trust in the institution with which they operate [58]. In applying theory, we might assume that because many public health officials work at various levels of government (e.g., municipal, provincial and federal), citizen’s trust in individual health experts would extend to trust in government [37] or indeed, be challenged if one did not trust government. Our data do not support theory in this case; rather, regarding pandemic communication, participants were accepting of health messaging and measures put into place to mitigate spread because they were generated by trusted sources of information (scientific sources and evidence-based information such as peer-reviewed literature and experts’ opinions), despite being communicated through government who were not trusted. This finding has important implications for public health communication because it emphasizes the need for public health and science to maintain public trust at a time when trust in government is declining.

The finding that the public, despite not trusting government, still trust individuals/institutions responsible for generating evidence may relate to a perceived separation on the part of the public between experts (broadly) and government. Managing a rapidly evolving pandemic like COVID-19 required (and still requires) government and policymakers to work with medical experts (virologists, epidemiologists, and public health professionals) and trusted agencies to craft and deliver evidence-based, consistent, and transparent public health messages [1]. However, it is unlikely that citizens, at a population level, have ever to this extent been required to consider if they trust their government given the unprecedented nature of the pandemic and related exposure to government representatives (e.g., the Prime Minister at the time held daily press conferences). This arguably created more opportunity for criticism and questioning of government action; however, studies indicate that because of the uncertainty of the virus, and perhaps public dependence on guidance, advice from experts (that is, scientists and medical experts) remained largely unquestioned [59]. As such, officials were likely to refer to experts’ epistemic authority when justifying unpopular measures and requesting their advice [59] but these experts, to the public, remained a separate entity. As such, their lack of trust in government did not factor into their trust in the messages being communicated through government.

Although beyond the aim of the present paper, we did consider our findings as they related to the political affiliation of participants. While trust in government was discussed by participants representing all political parties in Canada, including those that did not disclose their affiliation, left-leaning supporters were largely represented in our dataset. Unsurprisingly, those identifying as left-leaning were behind criticism of the government’s distribution of resources and perceived inequities in relation to service provision and public health communication. Of those who disclosed their political affiliation, it was also only left-leaning supporters that stated they trusted government messages because of who was behind the message. It is possible that right-leaning voices are captured in those unwilling to disclose their affiliation (37.5% of the sample), which may be because, at the time of data collection, the Liberal government was in power. However, we did face challenges in the recruitment of conservative supporters, and those representing the far right, which may relate to our role as scientists tied to a government institution [60]. As such, it cannot be assumed that individuals with right-wing ideology share the same sentiment regarding scientific or health information communicated through government, and this should be further explored with a more targeted sample.

## Conclusion

This novel qualitative study explored trust in government in response to the COVID-19 pandemic among a diverse group of Canadians. We found that trust in government was shaped by their perceptions of pandemic communication, as well as decision-making and implementation of pandemic measures. While trust in government does indeed matter, our data suggest that despite a lack of trust in government, participants were accepting of measures and messages as presented through government channels because the evidence generated to inform communication was thought to come from trusted sources. These data point to the importance of monitoring public trust in science and healthcare, both at an individual and institutional level, to ensure this trust is maintained. That is, while public trust in government needs to be (re)developed, the maintenance of trust in those generating evidence-based measures should be a priority. Indeed, understanding public behaviour [61] and the needs of citizens [9] during times of crisis is crucial for mitigating the COVID-19 pandemic. Research has suggested that governments need to use regular and rapid evaluation to assess the diverse public perceptions of risk and acceptance of preventive measures, as well as the public’s trust in government and in sources of information [61]. Collecting data using valid measures for the purpose of evaluating and monitoring trust in government and health organizations will permit a better understanding of public acceptance of government messaging and inform behavioural change measures, communication strategies, and policies in response.
